# Supplementary material for: The Power of Active Listening to Address Medication Non-Adherence During Care Transition: A Case Report of a Polypharmacy Patient with Type 2 Diabetes
Source: Pharmacy (Basel). 2025 Apr 30;13(3):64. doi: 10.3390/pharmacy13030064 (PMC12101230; doi:10.3390/pharmacy13030064)
Supplement: Supplementary file 1 [file pharmacy-13-00064-s001.zip › pharmacy-3537637-supplementary.pdf]

CARE CHECK LIST- The Power of Active Listening to Address Medication Non-Adherence During Care Transition: A Case Report of a Polypharmacy Patient with Type 2 Diabetes

Checklist Category	Checklist Item	Page	Comment
Title	The diagnosis or intervention of primary focus followed by the words “case report”.	1	
Key Words	2 to 5 key words that identify diagnoses or interventions in this case report (including 'case report').	1	
Abstract	Structured or unstructured abstract.	1	unstructured
Abstract	Introduction – What is unique about this case and what does it add to the scientific literature?	1	
Abstract	The patient’s main concerns and important clinical findings.	1	
Abstract	The primary diagnoses, interventions, and outcomes.	1	
Abstract	Conclusion – What are one or more “take-away” lessons from this case report?	1	
Introduction	Summarizes why this case is unique and may include medical literature references.	1-2	
Patient Information	De-identified patient-specific information.	2-3	
Patient Information	Primary concerns and symptoms of the patient.	2-3	
Patient Information	Medical, family, and psychosocial history including relevant genetic information.	2-3	
Patient Information	Relevant past interventions and their outcomes.	2-3	
Clinical Findings	Significant physical examination and important clinical findings.	N/A	Not relevant in this case report
Timeline	Historical and current information from this episode of care organised as a timeline.	2-3	In patient information
Diagnostic Assessment	Diagnostic methods (PE, laboratory testing, imaging, surveys).	N/A	Not relevant in this case report
Diagnostic Assessment	Diagnostic challenges	N/A	
Diagnostic Assessment	Diagnosis (including other diagnoses considered).	N/A	
Diagnostic Assessment	Prognostic characteristics when applicable.	N/A	
Therapeutic Intervention	Types of therapeutic intervention (pharmacologic, surgical, preventive).	N/A	
Therapeutic Intervention	Administration of therapeutic intervention (dosage, strength, duration).	3	Adapted to this case study: intervention = research interviews

Therapeutic Intervention	Changes in therapeutic interventions with explanations.	N/A	
Follow-up and Outcomes	Clinician- and patient-assessed outcomes if available.	4-5	Adapted to this case study as the Result section
Follow-up and Outcomes	Important follow-up diagnostic and other test results.	6	
Follow-up and Outcomes	Intervention adherence and tolerability.	6	
Follow-up and Outcomes	Adverse and unanticipated events.	6	
Discussion	Strengths and limitations in your approach to this case.	6-7	
Discussion	Discussion of the relevant medical literature.	6-7	
Discussion	The rationale for your conclusions.	6-7	
Discussion	Primary take-away lessons in a one paragraph conclusion.	6-7	
Patient Perspective	The patient should share their perspective on the treatment(s) they received.	4-7	Patient verbatim through the results
Informed Consent	The patient should give informed consent.	3;7	Present
